# In vivo response of AZ31 alloy as biliary stents: a 6 months evaluation in rabbits

**DOI:** 10.1038/srep40184

**Published:** 2017-01-13

**Authors:** Yang Liu, Shengmin Zheng, Nan Li, Huahu Guo, Yufeng Zheng, Jirun Peng

**Affiliations:** 1 Department of Materials Science and Engineering, College of Engineering, Peking University, Beijing 100871, China; 2 Department of Hepatobiliary Surgery, Peking University People’s Hospital, Beijing 100044, China; 3 Department of Surgery, Beijing Shijitan Hospital, Capital Medical University, Beijing 100038, China

## Abstract

Mg-based metallic materials have been making continuing progress as vascular stents. However, the research of Mg-based materials as non-vascular stents is still at its primary stage. AZ31 stents hereby were implanted into the common bile duct of rabbits for 6 months. The results revealed an existence of 93.82 ± 1.36% and 30.89 ± 2.46% of the original volume after 1 and 3 month, respectively. Whole blood tests indicated an inflammation decreasing to normal level after 3 month implantation. A benign host response was observed via *H&E* staining. Nonuniform corrosion at the two ends of the stents was observed and considered the results of flow or local inflammation. Moreover, the application of Mg-based materials for different stenting treatment were reviewed and compared. Esophagus was hypothesized most destructive, whilst blood vessel and bile duct considered similar and less destructive. Trachea and nasal cavity were thought to be mildest.

Mg and its alloys have been receiving continuously rising interests as potential absorbable coronary stents. The clinical trial on coronary stents fabricated from Mg alloys have made several progresses recently. The modified coronary stents based on AE21 and WE43 has evolved passing Lektron Magic, AMS-1, AMS-2, AMS-3 and DREAMS 1 G, with the composition, structure optimized and drug eluted^1^,^2^,^3^,^4^,^5^. The latest outcome of DREAMS 2 G demonstrated improved backbone design, higher radial force, higher bending flexibility and low lumen loss compared with its precursors during 6 month measurement, which made it a good candidate for current absorbable polymer stents^6^. More encouragingly, the magnesium coronary stents named Magmaris based on DREAMS 2 G from Biotronik Inc. announced CE approval in June 2016^7^. For Mg-based coronary stents, the blood composition and the flushing of the blood contribute to the different service condition compared with Mg-based orthopedic implants. Previous study has revealed increased corrosion rate of Mg alloys immersed in whole blood instead of SBF^8^. Besides, the high shear stress caused by flow increased the thickness of uniform corrosion product layer. Nevertheless, filiform corrosion greatly improved and quicken the biodegradation rate at high shear stress^9^,^10^.

Since composition and shear stress relevant with flow rate are critical for biodegradation of Mg and its alloys, the alteration of composition and lower flow rate may result in better corrosion resistance of the bare metal, implying the application in other non-vascular fluid environment. So far, besides blood vessel stents, the researches on Mg and its alloys as other kinds of non-vascular endoluminal stents still undergo their primary stage. Pure Mg has been reported to possess a corrosion rate of 0.01594 ml·cm^−2^·h^−1^ calculated from 10 days hydrogen evolution test in artificial saliva for potential esophageal stent application^11^. In this case, Mg has been reported as a relaxing factor of airway smooth muscles of rabbits^12^. Mg-3Y stent had been implanted into the trachea of canine model. The results revealed no adverse outcome of the adjacent cartilage structure after 8 weeks implantation^13^. Furthermore, an effective mechanical support for 3 months of JDBM alloy as tracheal stents in rabbits was reported^14^. However, for cartilage deficiency induced tracheomalacia, the stent should exist for 1 to 2 years, which lead to the evaluation of convenient way to apply multi-layer coatings^15^. Besides, regarding to Mg based alloys as intestinal stent, 100% extract of Mg-6Zn alloy had been reported to have significant higher apoptosis rate than 60% and 20% extracts, which implied that the intestinal epithelial cells are very sensitive to the corrosion rate of Mg alloys^16^. In addition, the *in vitro* test of Mg-4Y alloy, AZ31 alloy and pure Mg in artificial urine was reported. The results revealed good antibacterial which is needed for ureteral stent^17^.

Biliary stent is a kind of endoscopic non-vascular stent to treat benign biliary stricture besides traditional surgical methods. It provides options for those who cannot tolerate surgeries^18^. To date, the degradable polymer stent used to treat benign biliary stricture was reported to possess insufficient strength and displacement^19^. Meanwhile, human bile is a complex fluid system composed of inorganics, bile salts, bile acid, lecithin, cholesterol and some proteins^20^. Our previous *in vitro* study demonstrated only 1.87% weight loss after 60 days immersion in human bile^21^. Besides, previous study had demonstrated Mg alloys show no significant effects on the apoptosis of common bile duct epithelial cell^22^. However, the Mg-6Zn alloy was reported only 9% of the original weight remained after 3 weeks implantation in rabbit’s common bile duct (CBD)^23^. Besides, Mg-6Zn alloy was previous reported an increased *in vivo* corrosion rate as bone implants despite the alloy show good corrosion resistance *in vitro*^24^. Thus there is still a need for longer evaluation on a kind of Mg alloy to find the flexibility of Mg and its alloys as biliary stent. In this study, the first mid-term evaluation of biodegradable magnesium alloys as potential biliary stent has been reported. The AZ31 stent were implanted into the common bile duct (CBD) of rabbit. The biodegradation period was monitored and the tissues sections were evaluated.

## Results

### *In vivo* biodegradation

B-scan ultrasonography was utilized to examine the place of stent after 3 days implantation. Hyperechoic strip shadow was found in hepatic portal area, which indicated the stent was successfully implanted into the common bile duct of the rabbit (shown in Figure S1).

**Figure 1:**
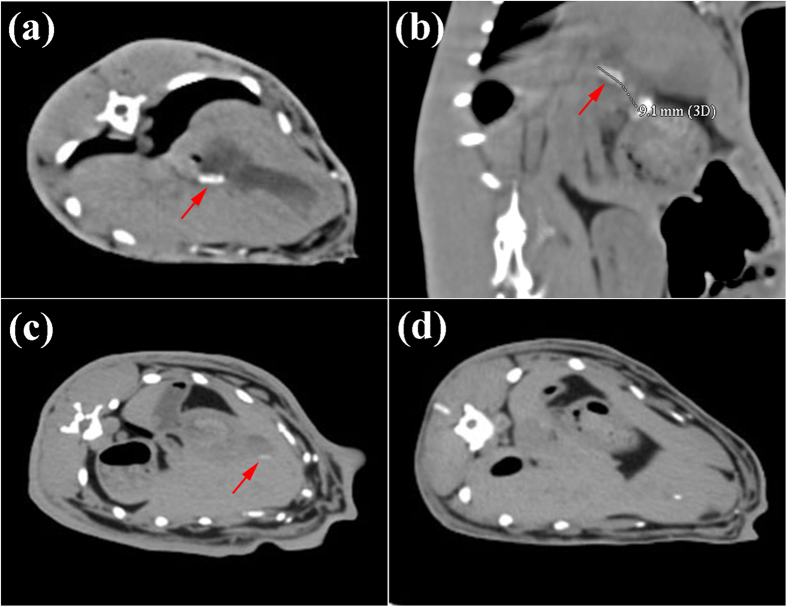
CT scan of the abdomen of the rabbits at 1, 3 and 6 month.

Figure 1 shows the CT scan of the abdomen of the rabbits at 1, 3 and 6 month. High density shadow was found in the abdomen 1 month post operatively (Fig. 1(a)). The CT scan in sagittal view (Fig. 1(b)) revealed a length around 9 mm of the stent, which indicated the stent maintained its shape and mechanical support after 1 month implantation. After 3 month, despite there is still a high density shadow observed in the abdomen (Fig. 1(c)), the shadow area of the stent in CT was much smaller compared with the results after 1 month implantation. CT scan after 6 month implantation showed that there was little apparent high density shadow found in the abdomen (Fig. 1(d)), which was thought to be the residue of implanted AZ31 stent.

### Characterization of retrieved stents

**Figure 2:**
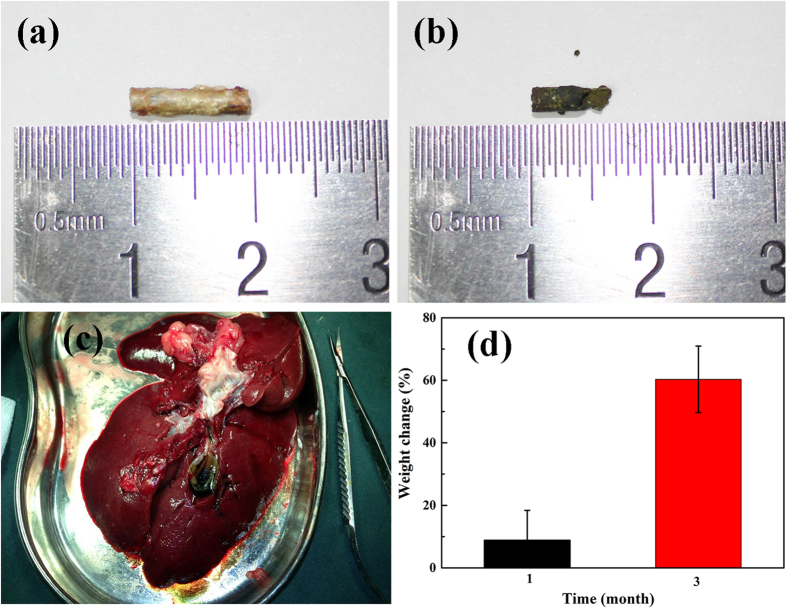
Condition of stent after (**a**) 1 month, (**b**) 3 month, (**c**) 6 month and (**d**) weight change after 1 and 3 month.

After CT scan at 1, 3 and 6 month, the rabbits were sacrificed and the bile duct was dissected. The stents were taken out, and the length and weight change were measured and calculated. As shown in Fig. 2, the stent remained a length around 9 mm after 1 month implantation, which is in good coherence with the CT results. Weight change before removal of corrosion products after 1 month implantation was 8.84 ± 9.55%. The stent maintained its morphology with the shape and edge well defined and the corrosion products distinguished. The stent in this study possessed a significant better corrosion resistance *in vivo* compared with Mg-6Zn, which was reported with 9% remained of the original weight after 3 weeks implantation^22^. However, the taken-out stent showed severe corrosion, with dark green corrosion products aggregated on the surface 3 months after surgery (Fig. 2(b)). Besides, the structure integrity was also damaged after 3 month implantation according to the digital image. The weight change before removal of corrosion products reached to 60.3 ± 10.6%. After 6 month implantation, no residues of the stent can be carried out. The stent fully degraded and small quantity of metallic residues were found, as well as little biliary sludge in the dissected biliary duct.

**Figure 3:**
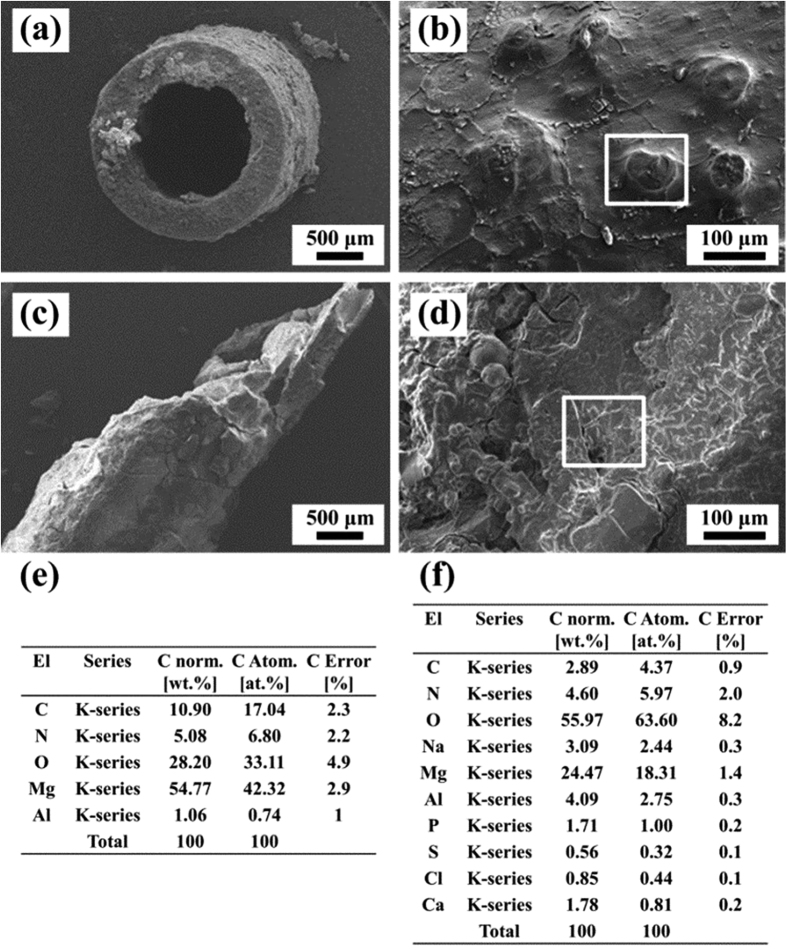
SEM images and coupled EDS results of retrieved stents after 1 and 3 month.

The surface morphologies as well as composition characterization of the taken-out stent at 1 and 3 month were shown in Fig. 3. The stent maintained an integrated structure and the margin of the stent remained clear for observation. Small groups of aggregation were seen on the surface. Besides, the EDS results revealed only C, O, N, Mg and Al were detected, indicating no complex reaction on the surface of the stent except the common biodegradation process of AZ31 and possibly some protein adsorption which induced the detection of element N after 1 month. However, the stent taken-out at 3 month after surgery showed severe corrosion. As shown in Fig. 3(c), the structure of the stent was damaged and some parts of the stent peeled off. Furthermore, the corrosion products aggregated on the surface seemed thicker and the composition of the corrosion layer was more complex compared with the results after 1 month implantation. Besides, after 3 month implantation, the content of Mg decreased but other elements like P, Ca, Na and S were detected, which implied there might be a secondary reaction after the corrosion of the AZ31 and the adsorption of proteins.

### High resolution 3D reconstruction

**Figure 4:**
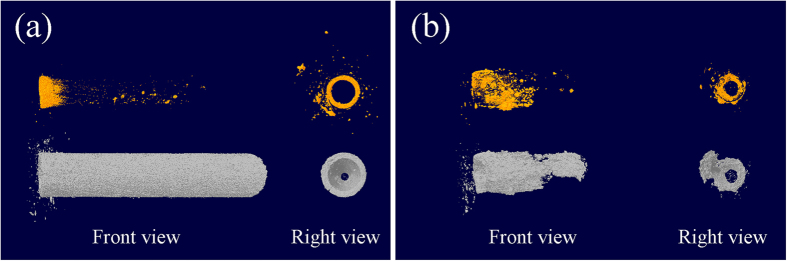
Micro-CT reconstruction of stent and corrosion products (**a**) after 1 month and (**b**) after 3 month. Note stent material is shown in grey and corrosion products in orange.

The taken-out stents were harvested for high resolution micro-CT reconstruction to clarify the shape of the stents, as well as the corrosion products and material remaining. The reconstructed materials as well as corrosion products are illustrated in Fig. 4. The volume of the remaining stent was calculated to be 93.82 ± 1.36%. The morphology of the stent remained integrated from both front view and right view (Fig. 4(a)). Besides, the corrosion products showed a preferred aggregates at the two ends of the stents. However, the stent underwent severe corrosion 3 months after operation, as shown in Fig. 4(b). The remained volume of the original stent was only 30.89 ± 2.46%. More importantly, a portion of the stent peeled off to disappear. The cross section of the stent deformed from the original roundness, suggesting a loss of mechanical integrity. As for the corrosion products, interestingly, preferred aggregation at one end more than the other was found, either. The nonuniform distribution of corrosion products may result from several aspects, such as nonunifom corrosion caused by flow, inflammation or different radial stress.

### Whole blood cell analysis

**Figure 5:**
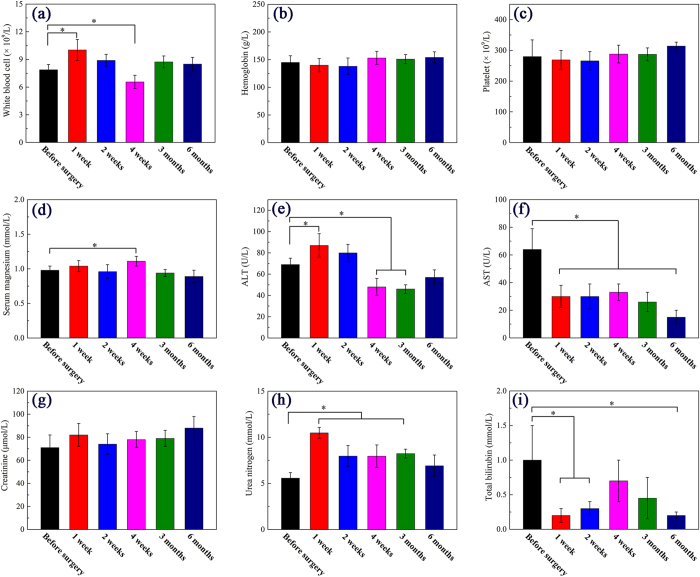
Whole blood analysis results before and after surgery, with “*” indicates significant difference compared with data gathered before surgery (P < 0.05).

Venous blood were monitored and analyzed, with testing results shown in Fig. 5. White blood cell concentration significantly increased 1 week after surgery (P < 0.05) but lowered down during the following period, indicating an inflammatory response after implantation, which disappeared 6 month after surgery (Fig. 5(a)). The white blood cell level at 6 month showed no significant difference compared with the results before surgery (P > 0.05). As for serum magnesium (Fig. 5(d)), the results suggested a significant increase 4 weeks after surgery (P < 0.05). After 1 month implantation, serum magnesium level decreased to normal. According to literature^25^, the normal range of white blood cells and serum magnesium in New Zealand White Rabbits are (5.2–16.5) × 10^9^/L and 0.65–1.33 mmol/L, respectively, suggesting the degradation rate is tolerable. Other kinds of parameters, such as Alanine aminotransferase (ALT) (Fig. 5(e)) and urea nitrogen (Fig. 5(h)) both underwent increase for a period of time after surgery. Nevertheless, 6 month after surgery, both the parameters back to normal. The Aspartate aminotransferase (AST) results (Fig. 5(f)) performed a continuing decrease of the value. According to literature, the AST value was reported to be in the range from 16.1 ± 6.8 U/L to 72.3 ± 12.0 U/L^26^. Thus the decrease of AST here was denoted no harm to the implanted rabbits. Overall, the whole blood cell analysis revealed a recovery process which is tolerable and less harmful *in vivo*.

### Histological evaluation

**Figure 6:**
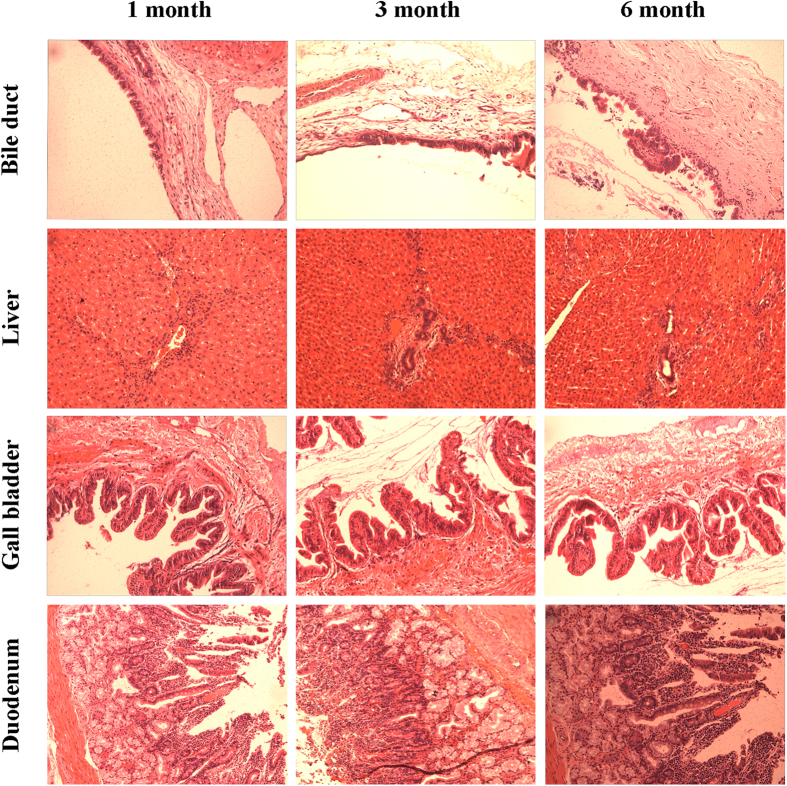
*H&E* staining of bile duct, liver, gall bladder and duodenum after 1, 3 and 6 month.

**Figure 7:**
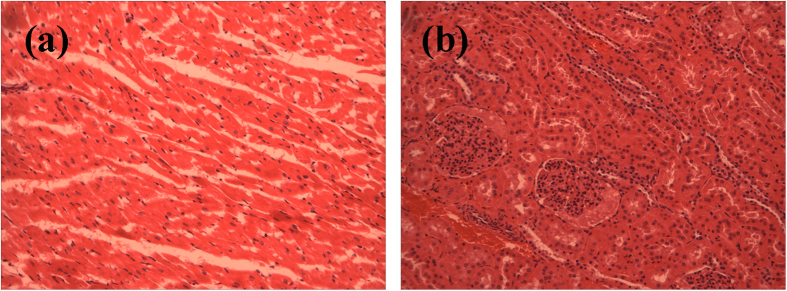
*H&E* staining of heat and kidney after 3 month implantation.

The *H&E* staining of bile duct, liver, gall bladder and duodenum after 1, 3 and 6 month are shown in Fig. 6. In some small parts, after 1 month implantation, the epithelium of bile duct revealed papillary hyperplasia, whilst the adventitia layer possessed fibrous hyperplasia, with infiltration with lymphocyte and eosinophils. After 3 and 6 month, the papillary hyperplasia still can be observed and some cell nucleus performed atypia, which may still be a results of inflammatory response. Meanwhile, the liver showed a recovery process after stent implantation. After 1 month, lymphocytes infiltration was observed as well as the inflammatory response at portal area. With time prolonging, after 3 month, the inflammatory response was reduced and cholangiectasis was seen only in 1 rabbit. No specific sign of abnormity was witnessed in liver after 6 month. As for gall bladder, the histological section showed no specific abnormality except for some calcified fragments at 1 and 3 month after surgery. The 6 month results revealed a papillary hyperplasia in gall bladder mucosa, similar to the results of bile duct. Furthermore, the histological section of duodenum revealed a mucosal chronic inflammation. Figure 7 shows the *H&E* staining of heart and kidney 3 month after implantation. As for cardiac muscular tissue, no obvious abnormality except punctate hemorrhage from large vessels and adipose tissue were observed. The kidney showed good arrangements of glomerulus and kidney tubules and no specific abnormality. Overall, the histological evaluation revealed acceptable *in vivo* biocompatibility of AZ31 stents.

## Discussion

The safety of implant devices is the primary issue concerned before clinical applications. The utilization of AZ31 as stent material may take a risk since there had been report on the toxicity of Al^27^,^28^,^29^. However, the LD_50_ of aluminum was reported to be 859 mg·kg^−1 ^27,30. Besides, the provisional tolerable weekly intake (PTWI) of Al was estimated to be 1 mg·kg^−1^ by the Joint FAO/WHO Expert Committee on Food Additives (JECFA)^31^. As for the present study, the calculated average weight change of AZ31 were 0.079 ± 0.014 mg·kg^−1^·d^−1^ or 0.035 ± 0.038 mg·kg^−1^·d^−1^, either by the weight change of 3 month and 1 month, respectively. Thus the corresponding estimated dissolved Al^3+^ were 0.00237 ± 0.00042 mg·kg^−1^·d^−1^ and 0.00105 ± 0.00114 mg·kg^−1^·d^−1^. Despite the fact the weight change is weighed before removal of corrosion products, there is still a significant gap between the reported tolerance limits of Al and the current report. Furthermore, the *H&E* staining of heart, liver and kidney show no adverse pathological changes. Our results revealed benign biosafety of AZ31 alloy as biliary stent.

Compared with the former application of Mg-6Zn as biliary stent material, the AZ31 revealed a much superior corrosion resistance^32^. However, the facts that about 60% weight loss/volume loss after 3-month-implantation and no residue found after 6-month-implantation suggest a better corrosion resistance *in vivo* is needed. Meanwhile, PLA, the bioabsorbable polymer counterpart used for biliary stent survival 6 month postoperatively^33^. For better improved corrosion resistance, a better understanding of the impact factors in the environment of the biliary implanted site is needed.

The human bile aids in emulsifying the large fat particles and absorption of the digested fat end products with the help of bile acid instead of any enzymes in the bile. Moreover, human bile works as transportation for excretion of waste from blood^20^. when it’s first secreted by liver and then after it has been concentrated in the gallbladder, the bile composition is as follows^20^. The liver bile consists of water 97.5 g/dl, bile salts 1.1 g/dl, bilirubin 0.04 g/dl, cholesterol 0.1 g/dl, fatty acid 0.12 g/dl, lecithin 0.04 g/dl, Na^+^ 145.04 mEq/L, K^+^ 5 mEq/L, Ca^2+^ 5 mEq/L, Cl^−^ 100 mEq/L and HCO_3_^−^ 28 mEq/L. The gallbladder bile consists of water 92 g/dl, bile salts 6 g/dl, bilirubin 0.3 g/dl, cholesterol 0.3 to 0.9 g/dl, fatty acid 0.3 to 1.2 g/dl, lecithin 0.3 g/dl, Na^+^ 130 mEq/L, K^+^ 12 mEq/L, Ca^2+^ 23 mEq/L, Cl^−^ 25 mEq/L and HCO_3_^−^ 10 mEq/L. As previously illustrated, the human bile is a complex environment at a pH of 7.8 and there are two kinds of human bile, with the primary difference be their water content. The gallbladder stored bile is more condensed and will release during digestion, otherwise the liver secreted bile exists in the human bile duct. The biliary stent in common bile duct in this study suffered from both kinds of bile. Besides, the bile composition, such as lecithin and bile acid, had been thought to help prevent the formation of crystalized gallstone^34^,^35^,^36^. In this case, the formation of CaCO_3_, insoluble Ca/P salts may be more difficult than any other body fluids without lecithin or bile acid^21^. Meanwhile, the soluble H_2_PO_4_^−^ had been reported to suppress the formation of corrosion pitting on WE43 alloy^37^. Thus the corrosion products and the corrosion mechanism can be different from the Mg-based stents for other kinds of applications.

Like Mg-based coronary stents, Mg-based biliary stents as well undergo corrosion fatigue process. The cyclic loading for coronary stents derives from arteries pulse, which can be as much as 40 million times per year^38^,^39^. However, for biliary stent, the cyclic loading results from the relaxation and contraction of the sphincter of Oddi. One will get his sphincter of Oddi relaxed to allow bile inflow into duodenum for assistance of digestion, and the sphincter of Oddi remains contracted over the rest of time. The contraction and relaxation of the sphincter of Oddi puts the bile duct in motion, which causes cyclic radial stress on the stents. Such motion also impact the bile flow rate. We interestingly found that the two ends of the stents possess different corrosion rate, resulting in different amount of corrosion product aggregation at the two ends (Fig. 4). Considering the implantation site is relative straight, the phenomenon may be due to the flow or local inflammation. Clinical applications had reported traditional metallic biliary stent failure^40^,^41^. Biodegradation process will lead to gradual loss of mechanical support. Especially for the biliary stent in human body, the lower part of human common bile duct turns an angle at the position between pancreas head and duodenum, which would also cause repeated bending for biliary stents^42^. The influence of corrosion fatigue and the corrosion fatigue behavior of Mg-based alloys as potential biliary stent materials should be further well concerned and investigated.

**Figure 8:**
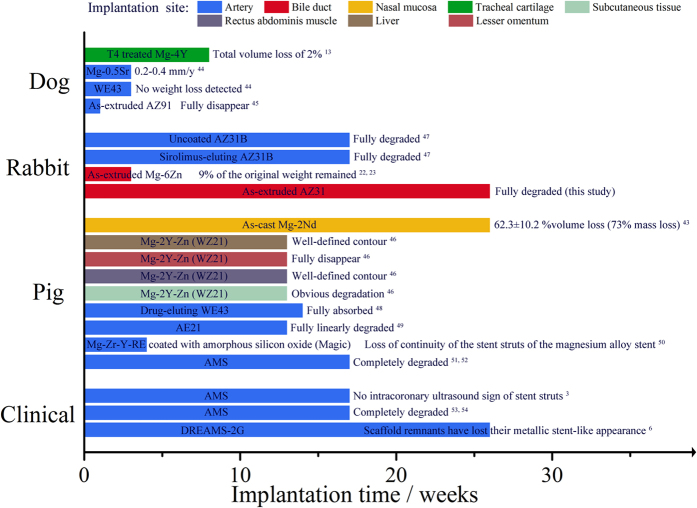
Summary diagram of *in vivo* corrosion performance of Mg and its alloys as stent for different applications.

Besides vascular stents, biliary stents, esophageal stents, colonic stents and pyloric/duodenal stents were utilized to treat different non-vascular stenosis. The application of Mg based alloys as stents is being expanded. Figure 8 summarizes the corrosion performance of Mg and its alloys for different target stent applications *in vivo*^3^,^6^,^13^,^22^,^23^,^43^,^44^,^45^,^46^,^47^,^48^,^49^,^50^,^51^,^52^,^53^,^54^.

The results revealed the corrosion rate ranges widely, given the consideration of different alloy composition, geometry and implantation environment. The adoption of Mg-3Y as tracheal cartilage ring only lost 2% of the original weight after 8 weeks implantation, despite fracture occurred around 5~6 weeks due to specific areas of high pressure^13^. Moreover, as-cast Mg-2Nd alloy in contact with nasal mucosa showed 63% volume loss after 180 days immersion, with the end direct contact with mucous membrane degraded faster than the other end indirect contact to membrane^43^. The corrosion media for Mg-2Nd was considered to be mucosa, mucus and air. Given the data gathered and the metabolism process in each position the stent is implanted, it is hypothesized as follow:

1. The tracheal cartilage and nasal environment provide a body environment less destructive than artery for magnesium alloys, due to the lower water contents and less organic and inorganic composition the stents get along with.
2. The biliary stents undergo a similar or a bit slower corrosion rate than the stents in artery due to its different composition compared with blood.
3. Esophagus may be more destructive to Mg and its alloys as stents than artery, due to the saliva swallow, intake of water and beverage and the occasional sour regurgitation.

The average corrosion performance of Mg-based alloys as coronary stent is considered to be 4 to 6 months. However, the comparative degree is unknown for now. To date, no publications directly compared the corrosion performance of same stents implanted for different usage. Besides, the composition of the alloys, the heat treatment of the alloy and the geometry of the device varies, which makes the comparison of the effects of the environment difficult. Thus a model metal is needed to assess the destructive influence and provide guidance for the corrosion degree of different implanting site, advising different absorbable metallic stents with different corrosion behavior for appropriate environment.

## Conclusions

In this study, AZ31 stents were implanted into the common bile duct of New Zealand Rabbits to assess the possibility as potential biliary stents for 6 month to see the mid-term feasibility and *in vivo* response. Micro-CT 3D reconstruction demonstrated a remaining volume of 93.82 ± 1.36% 30.89 ± 2.46% after 1 and 3 month, respectively. *H&E* staining and whole blood analysis confirmed the biosafety of AZ31 biliary stents. Flow or local inflammation was considered the cause of the nonuniform corrosion at the two ends of the stents. Moreover, the application of Mg-based alloys as non-vascular stents were reviewed and compared. Esophagus was considered most destructive, whilst blood vessel and bile duct considered similar and less destructive. Trachea and nasal cavity were thought to be mildest.

## Methods

### Material preparation

AZ31 alloy tubes were processed from as-extruded AZ31 alloy, with the external diameter of 2.2 mm, internal diameter of 1.8 mm and the length of 10 mm. Prior to the experiments, the tubes were chemical polished in polishing solution (20 ml glycerol, 2 ml hydrochloric acid, 3 ml nitric acid and 5 ml acetic acid), and then rinsed in ethanol and distilled water. After that, the tubes were weighted and then sterilized.

### Surgery

15 adult male New Zealand Rabbits with the weight of 2.5 ± 0.5 kg were used in this experiments. All animal experiments in this study followed the Care and Use of Laboratory Animals (issued by the Ministry of Science and Technology of the People’s Republic of China) and approved by the Ethics Committee of Peking University People’s Hospital (2012–43).

The rabbits were fasted for solids and liquids 8 hours before surgery. Rabbits were anesthetized with 3% intravenous sodium pentobarbital (30 mg/kg body weight) and then fixed as well as skin preparation and sterilized with iodine and ethanol. The middle of epigastrium was cut layer by layer, the gastrointestinal tract was shoved and the duodenum was lifted to find common bile duct, with the expanded intersection of common bile duct and duodenum visible. AZ31 stent was placed in the distal common bile duct via an off-angle 2 mm incision cut at the anterior wall of distal common bile duct. After that, the incision and epigastrium were sutured and closed. 5 × 10^4^ U/kg penicillin were intramuscular injected during the first 5 days post operation. At 1, 3 and 6 months, every 5 rabbits were sacrificed. Venous blood collected from the rabbits at 7 days, 14 days, 1 month, 3 month and 6 month post operation were evaluated for whole blood cell analysis.

### Ultrasonographic, radiographic and histological evaluation

B-scan ultrasonography were utilized after surgery to ensure the placement of AZ31 stent in the common bile duct. Computer Tomography (CT, Lightspeed VCT 64-MDCT, GE, United States) were conducted to observe the healing process before the removal of AZ31 stents at 1, 3 and 6 month before sacrifice. Besides, tissue samples from common bile duct, gall bladder, liver, duodenum, heart and kidney were collected and fixed in 10% formaldehyde for 24 hours. The samples then underwent dehydration, degreasing, embedding, section and *H&E* staining. The pathological change was observed through optical microscope.

### Characterization of retrieved stents

Stents were taken out at 1 and 3 month after surgery and rinsed in distilled water and dried in air. The length and weight change before removal of corrosion products were measured and calculated. Detailed surface morphologies and surface components were characterized via SEM coupled EDS (S-4800 Emission Scanning Electron Microscopy, HITACHI, Japan).

### Micro-CT 3D reconstruction

High-resolution micro-computed tomography (micro-CT; SkyScan 1172, SkyScan, Belgium) with 0.5 mm aluminum filter was adopted to assess and reconstruct the retrieval stents. The image pixel size utilized is 7.96 μm, with source voltage 59 kV and source current 124 μA. 3D reconstruction images and the fractional volume of stent material and corrosion products were generated by SkyScan software.

### Statistical analysis

Statistical analysis was performed with SPSS 18.0 for Windows software (SPSS Inc., Chicago, USA). One-way analysis of variance (ANOVA) followed by Tukey post hoc tests was used to statistically analysis all the data. A p-value < 0.05 was considered statistical significant difference.

## Additional Information

**How to cite this article**: Liu, Y. *et al*. In vivo response of AZ31 alloy as biliary stents: a 6 months evaluation in rabbits. *Sci. Rep.* **7**, 40184; doi: 10.1038/srep40184 (2017).

**Publisher's note:** Springer Nature remains neutral with regard to jurisdictional claims in published maps and institutional affiliations.

## Supplementary Material

Supplementary Information
