# Supplementary material for: In vivo response of AZ31 alloy as biliary stents: a 6 months evaluation in rabbits
Source: Sci Rep. 2017 Jan 13;7:40184. doi: 10.1038/srep40184 (PMC5234016; doi:10.1038/srep40184)
Supplement: Supplementary Information [file srep40184-s1.doc]

**In vivo response of AZ31 alloy as biliary stents: a 6 months evaluation in rabbits**

Yang Liu^a,1^, Shengmin Zheng^b,1^, Nan Li^a^, Huahu Guo^b^ , Yufeng Zheng^a,*^, Jirun Peng^c,**^

^a^ Department of Materials Science and Engineering, College of Engineering, Peking University, Beijing 100871, China

^b^ Department of Hepatobiliary Surgery, Peking University People's Hospital, Beijing 100044,China

^c^ Department of Surgery, Beijing Shijitan Hospital, Capital Medical University, Beijing 100038,China

**
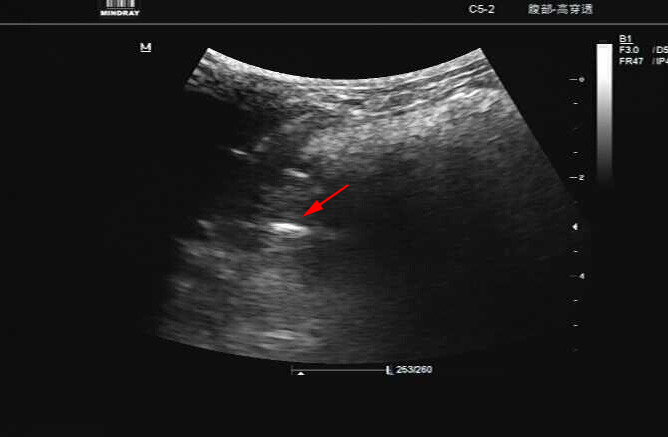
**

**Supplementary Figure S1** B-scan ultrasonography of the stent after 3 days implantation.
